# Valentino's Syndrome in the Era of Advanced Imaging and Minimally Invasive Surgery

**DOI:** 10.7759/cureus.74735

**Published:** 2024-11-29

**Authors:** Ana K Andrade, Clara Sampaio, Anjum Dhanani

**Affiliations:** 1 General Surgery, Unidade Local de Saúde de São José, Lisbon, PRT

**Keywords:** acute abdominal pain, acute abdominal surgery, appendectomy, appendicitis, complicated peptic ulcer disease, laparoscopic appendectomy, laparoscopic surgery, perforated peptic ulcer, valentino's syndrome

## Abstract

Valentino's syndrome is a rare but potentially lethal differential diagnosis for acute appendicitis. We herein present the case of a 22-year-old male patient who presented to the emergency department with acute abdominal pain. Clinical suspicion of acute appendicitis was corroborated by analytical and imaging findings. However, surgical exploration revealed a perforated peptic ulcer. A high degree of suspicion during preoperative and intraoperative management is important in recognizing and treating this condition, which carries significant morbidity and mortality risks.

## Introduction

Acute appendicitis is one of the most common conditions encountered in emergency settings, often presenting with right lower quadrant pain [1-3]. Clinical evaluation and diagnostic workup are vital as the differential diagnosis is extensive. Peptic ulcer disease on the other hand, while prevalent, rarely manifests as a complication such as bleeding or perforation. Valentino's syndrome, a rare but potentially life-threatening condition, occurs when a perforated peptic ulcer mimics acute appendicitis, clinically, biochemically, and radiologically, as the fluid from the perforation migrates into the right paracolic gutter leading to localized peritonitis in the right lower quadrant which can cause pain in the right iliac fossa, elevation of inflammatory markers, and fat stranding on radiographic imaging [1-7]. This entity requires surgical resolution, and correct and timely diagnosis is crucial as a delay in treatment can increase its morbidity and mortality [3]. Advances in radiological imaging have improved the diagnostic accuracy and outcome of this pathology, historically diagnosed during surgery [3]. Minimally invasive surgery plays a pivotal role as it offers both diagnostic and therapeutic advantages. 

## Case presentation

A 22-year-old man presented to our emergency department with abdominal pain that began two hours prior to admission. The pain initially localized in the periumbilical region and later migrated to the right lower quadrant. The patient denied any history of fever, bowel habit changes, urinary symptoms, nausea, or vomiting. His medical and surgical histories were unremarkable. 

On examination, the patient was hemodynamically stable. Physical findings included diffuse abdominal pain with tenderness in the right iliac fossa. Blumberg's and Rovsing's signs were negative. 

Laboratory tests revealed a neutrophilic leukocytosis of 19.410 cells/μl (4.5-11.0) and 86.4% neutrophils (40-75), without the elevation of C-reactive protein levels. 

**Figure 1:**
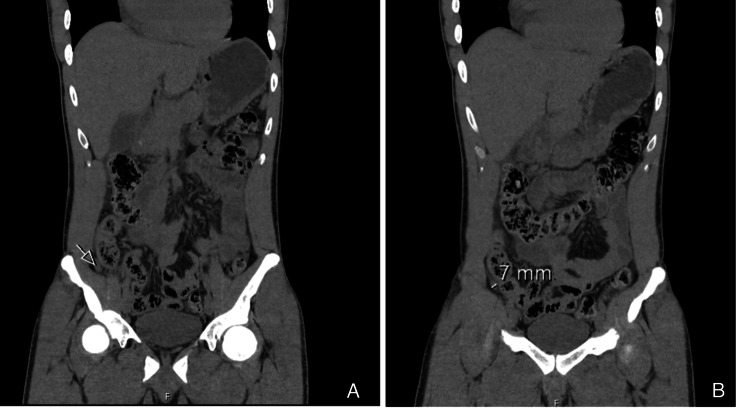
Coronal CT scan showing inflammatory changes in the right iliac fossa (A) and a 7 mm appendix (B)

A CT scan performed four hours after the beginning of symptoms showed a 7 mm appendix with adjacent fat stranding and free fluid in the pelvic region and paracolic gutter (Figure 1), with no signs of pneumoperitoneum or other evidence of perforation; thus, an acute appendicitis was presumed. 

**Figure 2:**
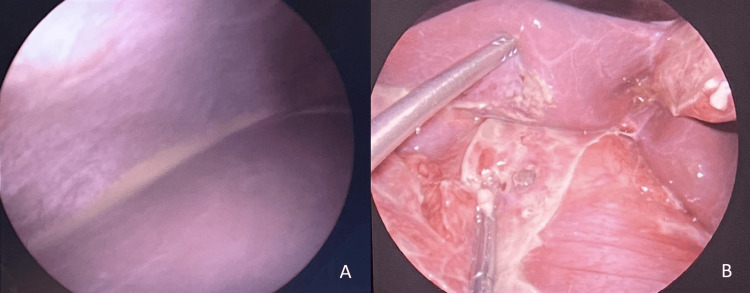
(A) Purulent peritonitis during laparoscopy. (B) Fibrinopurulent membranes and pyloric perforation

The patient was taken for laparoscopic appendectomy within 24 hours of admission. During surgery, moderate purulent peritonitis was found, and turbid fluid in the right paracolic gutter led to further exploration of the abdomen with findings of fibrinopurulent membranes along the liver bed (Figure 2A). Immature adhesions of the stomach to the gallbladder, liver, and round ligament were lysed, and an approximately 5 mm pyloric perforation was identified (Figure 2B). A primary repair of the perforation with an absorbable and an omental patch (Graham patch technique) followed by appendectomy was performed. Extensive abdominal lavage was done, and a drain was placed in the subhepatic region. 

Postoperatively, the patient had an uneventful recovery. Intravenous antibiotics were administered, and the nasogastric tube was removed on the second postoperative day. Oral intake was resumed gradually, and the drain was removed on postoperative day 4. The patient was discharged on day 5. He was treated for *Helicobacter pylori* and a routine upper endoscopy was performed. Follow-up assessments were unremarkable. 

## Discussion

Valentino's syndrome was first described in 1926, when Rudolph Valentino, a renowned American actor, underwent an appendectomy, which had an unfavorable evolution as it was actually an undiagnosed perforated peptic ulcer that later progressed to peritonitis, secondary multiorgan failure, and ultimately his death [1,2,4,5,7].

Perforation is a rare complication of peptic ulcer disease, occurring in approximately 10% of cases, and it is considered a surgical emergency. *Helicobacter pylori* and non-steroidal anti-inflammatory drug (NSAID) use are the most common causes of peptic ulcer perforation [2,4,5,7]. It has the highest mortality rate of any complication of ulcer disease approaching 15%; however, it has decreased due to increased surgical proficiency [1,4].

Few cases of Valentino's syndrome have been reported in the literature and its incidence and prevalence remain unknown. 

Despite Valentino's syndrome being a rare presentation of peptic ulcer disease, it is highly relevant to be taken into consideration as it might present as a life-threatening condition if not treated correctly and promptly. Therefore, high suspicion is required as a delay in diagnosis and treatment can result in potential morbidity and mortality [2,3].

Abdominopelvic CT is considered the imaging method of choice and according to literature is the most commonly used method to make the diagnosis in over 70% of the cases. Findings include gastric or duodenal wall thickening, adjacent fat stranding or inflammatory changes, and occasionally visualization of the perforation site [3,7].

The diagnosis however is often made intraoperatively [6]. Intraoperative findings of a normal appendix and fibrinous or bile-stained exudates along the right paracolic gutter should raise the suspicion for a perforated ulcer and demand a thorough inspection of the stomach and duodenum for sites of perforation [5,6]. In the case of gastric ulcer, biopsies are recommended to rule out malignancy, and eradication for *Helicobacter pylori *should be performed [3].

## Conclusions

Perforated peptic ulcer disease poses a significant diagnostic challenge due to its variable presentation. Valentino's syndrome is a rare but important condition to consider when managing acute abdominal pain. Radiological imaging and minimally invasive surgery play key roles in the accurate diagnosis and treatment of this pathology. Our aim is to raise awareness of this condition as it is essential for emergency physicians, radiologists, and surgeons to consider this entity in order to reduce its potential morbidity and mortality. Minimally invasive surgery provides a significant advantage over open surgery offering enhanced diagnostic and therapeutic benefits. 
